# Mitophagy in gastrointestinal tumors: mechanisms and new targets for immunotherapy

**DOI:** 10.3389/fonc.2025.1717138

**Published:** 2026-01-02

**Authors:** Tao Zhang, Zhetan Ren, Bowen Tang, Ru Man, Lin Wang, Qingyan Wang, Jirun Peng, Yongduo Yu

**Affiliations:** 1 Liaoning University of Traditional Chinese Medicine, Shenyang, China; 2 Department of General Surgery, Beijing Shijitan Hospital, Capital Medical University, Beijing, China; 3 Second Affiliated Hospital, Liaoning University of Traditional Chinese Medicine, Shenyang, China

**Keywords:** drug resistance, gastrointestinal tumors, immune escape, immunotherapy, mitophagy

## Abstract

Gastrointestinal tumors (GITs), particularly gastric and colorectal cancers, are the leading causes of cancer-related deaths worldwide. Despite advances in screening technologies and the continuous development of treatments, which have improved early diagnosis and therapeutic interventions, the morbidity and mortality rates remain high, presenting a significant challenge to global public health. While existing treatments can extend patient survival to some degree, they are often accompanied by substantial side effects. In recent years, immunotherapy has yielded positive outcomes for some patients. However, the emergence of immune escape mechanisms has hindered treatment effectiveness. As a result, there is an urgent need for new therapeutic strategies that can address the limitations of current approaches. mitophagy, a key cellular process, has gained significant attention in cancer research. It plays an essential role in maintaining cellular energy balance and metabolic stability, and is intricately linked to crucial biological processes such as drug resistance, metastasis, invasion, and the tumor microenvironment. This article aims to provide a comprehensive review of the mechanisms underlying mitophagy, examining its role in gastrointestinal cancers, particularly in relation to cellular metabolism, apoptosis, drug resistance, metastasis, invasion, and the tumor microenvironment. Additionally, it will explore the potential of mitophagy as a therapeutic target and address current clinical challenges. It is hoped that this research will offer new insights and directions for the treatment of GITs.

## 1 Introduction

Gastrointestinal tumors (GITs) refer to malignant tumors that occur in the gastrointestinal organs of the digestive system. According to the World Health Organization, GITs are among the most common malignancies globally, with over one million new diagnoses each year (1, 2). In 2021, approximately one million new cases of gastric cancer were reported worldwide, with nearly 780,000 deaths; the incidence of colorectal cancer continues to rise globally, particularly in economically developed regions (3, 4). Despite continuous advancements in medical technology, the incidence and mortality rates of GITs remain alarmingly high (5–7). Although GITs have been extensively studied, current treatment options still have significant limitations. Presently, the treatment of GITs relies mainly on surgery, chemotherapy, radiotherapy, and targeted therapy (8). While these treatments have extended patient survival to some extent, they are often accompanied by considerable side effects (9).

In recent years, immunotherapy has emerged as a promising new treatment strategy, showing potential in certain tumor subtypes, particularly those with high microsatellite instability (MSI-H) and deficient mismatch repair (dMMR), where it has demonstrated good clinical efficacy (10–12). However, the inconsistent effectiveness of immunotherapy, especially due to immune escape phenomena, has limited its clinical success (13).

In this context, mitochondrial autophagy, also known as mitophagy, has drawn increasing attention as a key mechanism regulating cellular immune responses. By selectively degrading damaged mitochondria, mitophagy plays a crucial role in cell survival, proliferation, and immune response (14). In particular, in tumor cells, mitophagy plays a significant role in cell growth, drug resistance, and immune evasion (15, 16). Aberrant regulation of mitophagy is closely associated with the initiation and progression of GITs, making it a hot topic in the research of novel immunotherapeutic strategies (17, 18). The enormous potential of mitophagy in tumor cells offers new hope for advancing immunotherapy.

Although some studies have explored the role of mitophagy in cancer, particularly in areas such as metabolism, drug resistance, and cell death, a comprehensive review of mitophagy mechanisms in GITs remains lacking (19, 20). Moreover, how mitophagy contributes to immune evasion, modulates the tumor microenvironment, and its potential application in immunotherapy remains underexplored. Therefore, this paper aims to systematically summarize the role of mitophagy in GITs, focusing on its involvement in tumor cell generation, progression, apoptosis, and drug resistance, while exploring its potential as a novel target for immunotherapy.

## 2 Current research status of GITs

GITs are a severe form of malignant tumors. Despite advancements in early screening techniques and treatment options in recent years, the high incidence and mortality rates of GITs still make them a significant challenge in global cancer prevention and control efforts. Currently, the main therapeutic strategies for GITs each have their own advantages and limitations. While surgical resection offers curative potential in early-stage GIT patients, its efficacy is limited for advanced or metastatic cases. Chemotherapy and radiotherapy can alleviate symptoms and extend survival to some extent, but they are accompanied by significant side effects and issues with drug resistance (21–23). This is particularly concerning as patients’ quality of life is often negatively impacted during the course of treatment (24, 25).

Targeted therapies, as a relatively new treatment approach, can act on specific molecular targets. However, the development of resistance and inter-patient variability hinder the widespread applicability of these treatments. Immunotherapy has shown promising clinical efficacy in certain GIT cancer subtypes, such as MSI-H and dMMR patients, yet immune escape mechanisms limit its broader application.

It is noteworthy that mitophagy regulation, as a potential adjunctive therapeutic strategy, is still in the research phase. However, it holds promise in overcoming tumor cell resistance to conventional treatments and modulating immune functions, which could lead to novel therapeutic breakthroughs. **Table 1** summarizes the common therapeutic approaches for GITs, their mechanisms of action, and the associated clinical limitations.

**Table 1:** Common therapeutic approaches for gastrointestinal tumors.

Treatment method	Advantages	Limitations	References
Surgical Resection	Effective for early-stage GIT cancers; offers the potential for complete tumor resection and cure.	Applicable only for localized tumors; difficult to treat advanced or metastatic cases.	(26)
Chemotherapy	Effective for advanced GIT cancers; alleviates symptoms and extends survival.	Significant side effects such as nausea, vomiting, immunosuppression, and bone marrow suppression; gradual emergence of chemotherapy resistance reduces efficacy.	(21, 22)
Radiotherapy	Effective for locally advanced GIT cancers; can alleviate pain and symptoms.	Potential side effects include radiation-induced enteritis; limited efficacy for metastatic tumors.	(23)
Targeted Therapy	Targets specific molecules (e.g., EGFR, VEGF) to inhibit tumor growth, with relatively fewer side effects.	Resistance develops over time, and not all patients respond to targeted drugs; treatment must be personalized based on individual differences.	(24, 25)
Immunotherapy	Significant efficacy in GIT cancer subtypes with MSI-H and dMMR; improves patient survival rates.	Immune escape mechanisms in some patients limit the widespread applicability of immunotherapy.	(27, 28)

## 3 The basic concept and mechanism of mitophagy

Autophagy is a fundamental catabolic process through which cells eliminate damaged organelles and misfolded proteins via the lysosomal degradation pathway. Mitophagy represents a selective subtype of autophagy that specifically targets mitochondria. As the primary energy-producing organelles, mitochondria generate the majority of cellular ATP, and the preservation of their functional integrity is essential for cellular viability (29). Through the recognition and removal of damaged or dysfunctional mitochondria, mitophagy sustains cellular energy homeostasis and metabolic equilibrium, while mitigating the accumulation of oxidative stress (30).

Currently, mitophagy is thought to proceed primarily through two distinct pathways: mitochondrial membrane receptor–mediated signaling and the PINK1/Parkin pathway. In the receptor-dependent mechanism, proteins such as BNIP3, NIX, and FUNDC1 located on the outer mitochondrial membrane directly interact with the autophagy-associated protein LC3, enabling damaged mitochondria to be selectively sequestered into autophagosomes. This process is relatively straightforward and is commonly activated under hypoxic or cellular stress conditions (31, 32).

**Figure 1:**
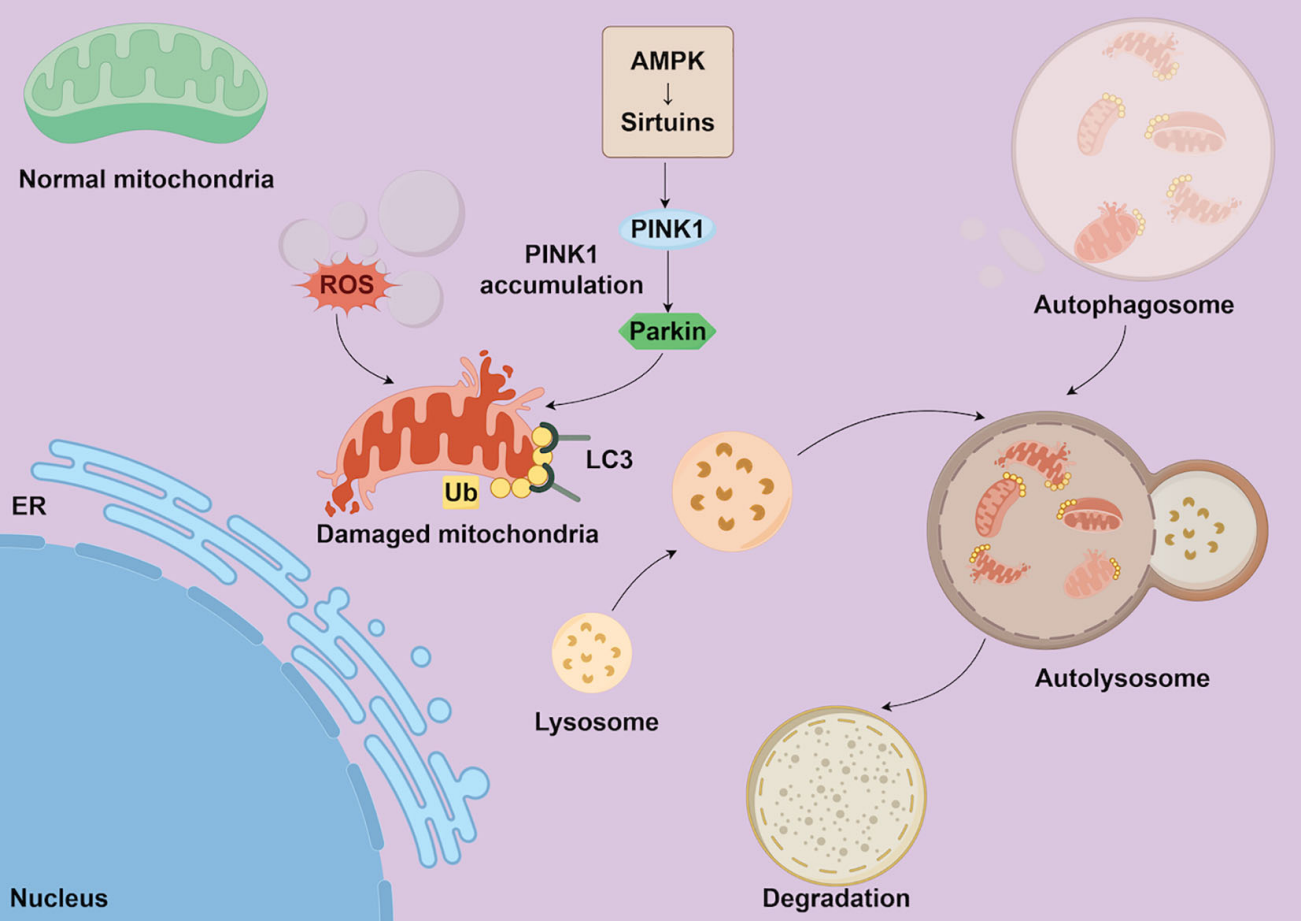
The mechanism of mitophagy. Legend of **Figure 1**: This figure illustrates the mechanism of mitophagy through the PINK1/Parkin pathway. Damaged mitochondria are recognized through the accumulation of ROS, which activates PINK1. PINK1 then recruits Parkin, marking the damaged mitochondria for degradation. LC3 plays a role in the formation of autophagosomes, which eventually fuse with lysosomes for mitochondrial degradation. This process helps maintain cellular energy metabolism and overall cell function.

The PINK1/Parkin pathway represents the more classical mechanism. When mitochondrial membrane potential declines, PINK1 is unable to translocate into the matrix and consequently accumulates on the outer membrane, where it recruits the E3 ubiquitin ligase Parkin. Parkin ubiquitinates multiple outer mitochondrial membrane proteins, thereby facilitating the recruitment of autophagy receptors such as p62, NDP52, and OPTN to mediate autophagosome formation. Through this cascade, cells identify and remove dysfunctional mitochondria, thereby preventing excessive accumulation of reactive oxygen species and preserving mitochondrial quality control and metabolic homeostasis (33, 34). As shown in **Figure 1**, mitophagy occurs through the PINK1/Parkin pathway, where damaged mitochondria are identified and removed by the autophagic machinery.

Furthermore, mitophagy is intricately regulated by multiple metabolic signaling pathways, including mTOR, AMPK, and the Sirtuin family, which collectively sense cellular energy status and stress levels. AMPK activation generally promotes the initiation of autophagy by suppressing mTORC1 activity, whereas Sirtuin-mediated deacetylation modulates the function of key autophagy regulators (such as PGC-1α and FOXO3a), thereby indirectly influencing the dynamic balance between mitochondrial biogenesis and clearance (35–37). Overall, mitophagy serves not only as a quality control mechanism for the removal of damaged mitochondria but also as a pivotal regulatory node linking energy metabolism, redox homeostasis, and immune responses.

## 4 Mitochondrial isolation and autophagy assessment methods

**Figure 2:**
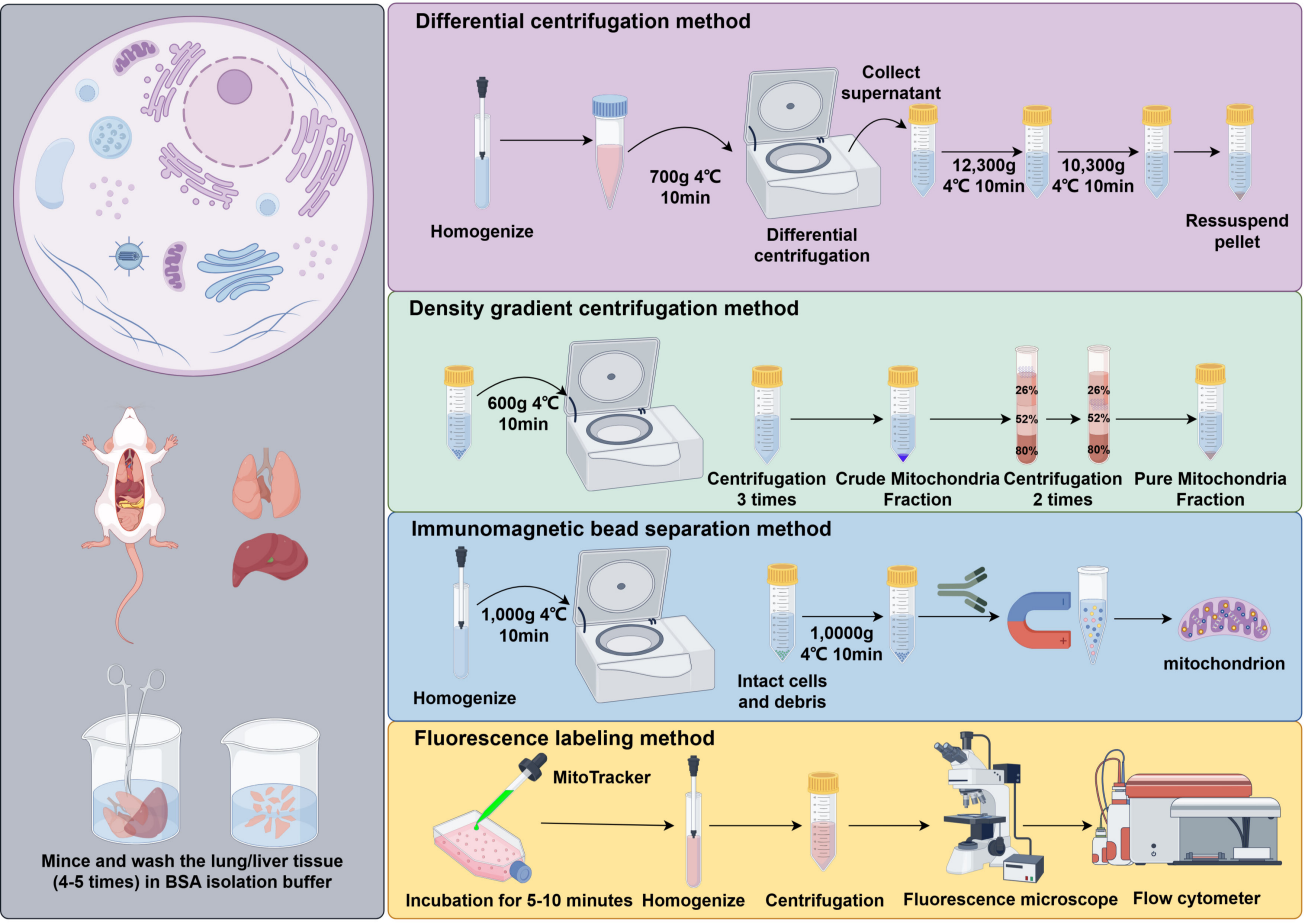
Methods for mitochondrial isolation. Legend of **Figure 2**: This figure summarizes four commonly used methods for mitochondrial isolation: differential centrifugation, density gradient centrifugation, immunomagnetic bead separation, and fluorescence labeling. Each method has its own advantages in terms of purity and specificity, depending on the experimental needs.

Mitochondria, as critical organelles, directly influence cellular homeostasis and disease development through their structural and functional integrity. Accurate investigation of mitochondrial function and associated autophagy processes requires the isolation of high-purity, structurally intact mitochondrial preparations. Currently, commonly employed isolation techniques include differential centrifugation, density gradient centrifugation, immunomagnetic bead-based separation, and fluorescent labeling. As shown in **Figure 2**, various methods for mitochondrial isolation are commonly employed to purify mitochondria for further analysis.

Differential centrifugation is widely employed for isolating mitochondria from routine cell or tissue samples due to its simplicity and high reproducibility, although its purity and structural integrity are relatively limited (38). Density gradient centrifugation can further enhance resolution and purity, making it suitable for the more precise separation of mitochondrial subpopulations (39). Immunomagnetic bead-based separation utilizes specific antibodies for rapid and selective enrichment, but it is costly and heavily dependent on antibody quality (40). Fluorescent labeling is frequently applied in live-cell experiments to dynamically monitor changes in mitochondrial morphology and distribution, although it may partially interfere with normal mitochondrial function (41). Consequently, researchers must select the most appropriate isolation method according to the study objectives, sample type, and experimental conditions.

The evaluation of mitophagy encompasses a variety of experimental approaches. Fluorescence microscopy can visually capture the interaction between mitochondria and autophagosomes and remains one of the most commonly employed observation techniques, although its quantitative capacity is limited (42). Western blot analysis of autophagy-related markers (such as LC3 and P62) provides robust quantitative assessment of autophagic activity (43). Flow cytometry is valued for its high throughput and sensitivity, making it suitable for population-level functional analyses (44). Electron microscopy allows direct visualization of mitochondrial ultrastructural changes and is regarded as a critical method for confirming autophagy, though it requires stringent experimental conditions and advanced technical skills (45). Additionally, measurements of oxygen consumption rate (OCR) or cellular ATP content are frequently employed to indirectly evaluate mitochondrial functional status.

In summary, different mitochondrial isolation and autophagy assessment techniques possess distinct characteristics in terms of sensitivity, specificity, and operational complexity, and their selection should be carefully considered based on the research objectives and experimental requirements. For structural and functional analyses, density gradient centrifugation and electron microscopy are particularly suited for detailed investigation, whereas fluorescent labeling and flow cytometry offer advantages for dynamic monitoring or high-throughput screening. Immunomagnetic bead-based separation is well-suited for targeted studies of specific cell populations or mitochondrial subpopulations. The judicious selection and combination of multiple methods can not only enhance the reliability of experimental results but also provide a more comprehensive understanding of the relationship between mitochondrial function and autophagy regulation.

## 5 Mechanisms of mitophagy in GITs cells

**Figure 3:**
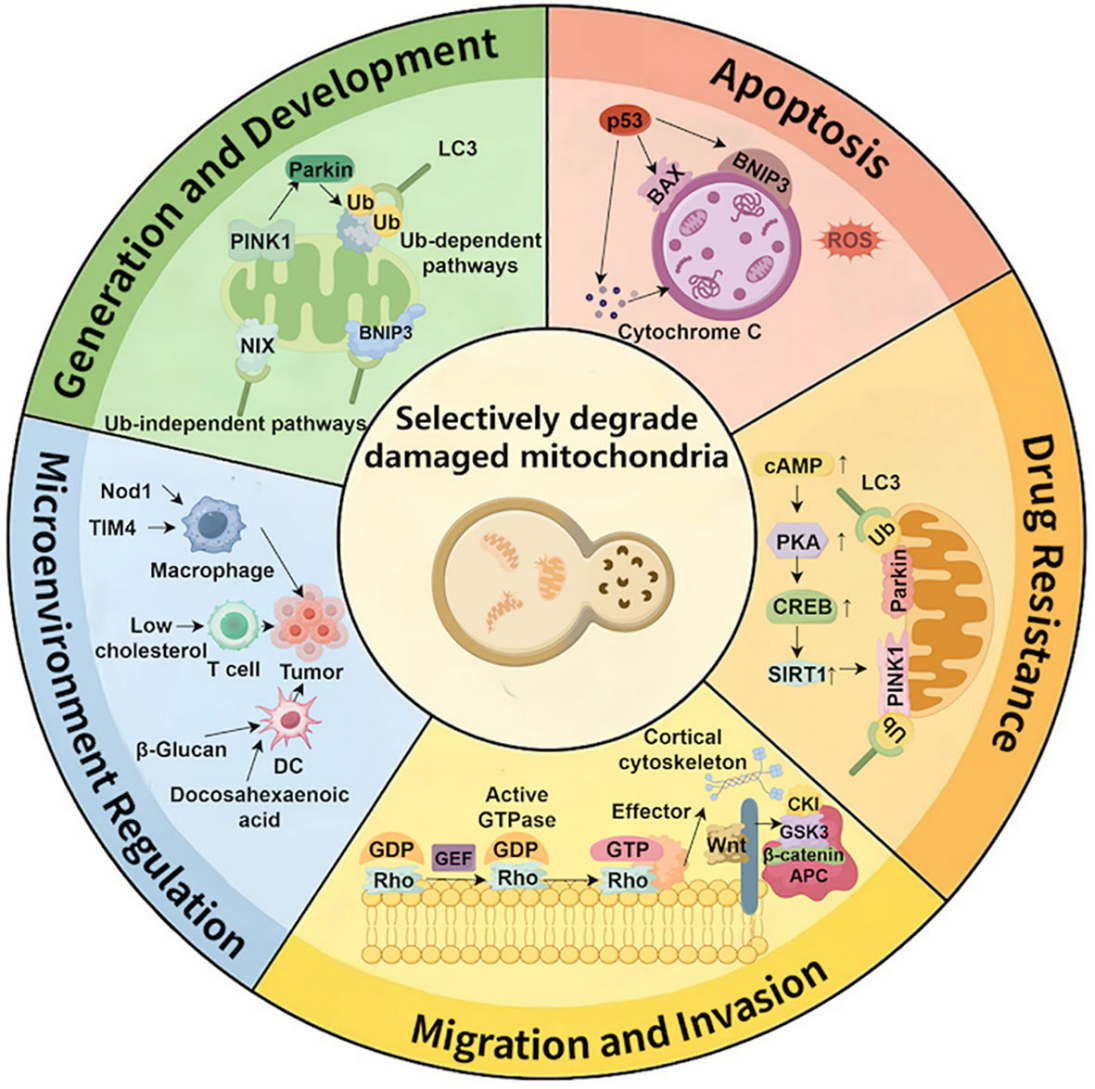
The role of mitophagy in various cellular processes. Legend of **Figure 3**: This figure illustrates the diverse roles of mitophagy in regulating several cellular processes. It shows how mitophagy participates in cell generation and development, apoptosis, drug resistance, microenvironment regulation, and tumor migration/invasion.

Mitophagy plays a pivotal role in maintaining cellular homeostasis, energy metabolism, and immune responses, which are crucial for various aspects of tumor cell behavior, including growth, migration, invasion, drug resistance, and immune evasion. By regulating redox balance, cellular metabolism, and interactions within the tumor microenvironment, mitophagy determines the survival capacity of tumor cells. As shown in **Figure 3**, mitophagy plays a crucial role in regulating key cellular processes, including cell generation, apoptosis, drug resistance, tumor migration, and microenvironment modulation.

### 5.1 Cell generation and development

The generation and development of GITs cells are often accompanied by metabolic changes. Mitophagy contributes to maintaining metabolic balance in tumor cells by removing damaged mitochondria, reducing the accumulation of ROS, and preventing cell death caused by mitochondrial dysfunction (46, 47). Mechanistically, when mitochondria are damaged, PINK1 accumulates and recruits Parkin, initiating the mitophagy process. Mitophagy not only removes damaged mitochondria but also maintains ATP supply and regulates ROS levels, promoting tumor cell proliferation in hypoxic and nutrient-deprived environments (48, 49). BNIP3 and NIX are key regulators of mitophagy. By binding to autophagosomes or interacting with other intracellular structures, they facilitate the fusion of damaged mitochondria with autophagosomes (50–52).

Additionally, mitophagy regulates fatty acid oxidation pathways, reduces endogenous ROS production, sustains energy supply, and supports tumor cell proliferation (53, 54). Inhibition of mitophagy significantly suppresses tumor growth and increases ROS levels, underscoring the importance of mitophagy in tumor progression (55). Clinical studies have also found that enhanced mitophagy is closely associated with tumor proliferation in colorectal cancer patients. This mechanism helps maintain cellular metabolic stability and energy balance, supporting tumor cell growth (56–58).

### 5.2 Cell apoptosis

Apoptosis is a core mechanism for maintaining tissue homeostasis and eliminating damaged or potentially malignant cells, and it plays an important role in suppressing cancer during the early stages of tumor development. However, many tumors suppress apoptosis by downregulating death-receptor pathways, upregulating anti-apoptotic proteins, or inhibiting p53 signaling, thereby acquiring the ability to proliferate continuously and evade immune surveillance (59). In gastrointestinal tumors, the relationship between mitophagy and apoptosis is particularly complex, and its dysregulation often directly influences cell fate.

Current studies have shown that mitophagy inhibits oxidative stress-induced cell death by reducing ROS levels (60). However, in certain cases, the inhibition of mitophagy may result in the accumulation of damaged mitochondria, which in turn activates endogenous stress signals such as AMPK and p53, prompting cells to enter an apoptotic state (61–63). When cells are in a low-energy state, the AMPK pathway is activated, inhibiting the mTORC1 signaling pathway, which enhances mitophagy and prevents cell death due to energy deficiency (64–66). p53 can upregulate BNIP3, promoting mitophagy to remove damaged mitochondria and prevent apoptosis (67, 68). Additionally, p53 regulates mitochondrial outer membrane permeability, releasing cytochrome C and modulating both mitophagy and the Caspase-3 pathway to maintain cellular stability (69, 70).

In a mouse model of breast cancer, inhibition of mitophagy leads to increased ROS levels, activation of p53, and induction of tumor cell apoptosis (71). In another experiment, p53 promoted mitophagy by upregulating BNIP3 while reducing the accumulation of ROS in mitochondria (72). Clinical observations have indicated that in breast cancer patients, tumor cells with higher levels of mitophagy exhibit longer survival, suggesting that mitophagy helps inhibit tumor cell apoptosis and improves drug resistance (73, 74).

### 5.3 Cellular drug resistance

Tumor cell drug resistance remains a significant challenge in cancer treatment. Mitochondria supply energy to tumor cells, prevent cellular dysfunction, and contribute to the enhancement of drug resistance. SIRT1, a deacetylase and a member of the sirtuin family, is widely distributed in cells, particularly within the nucleus and cytoplasm (75). Mitophagy can upregulate SIRT1 expression through the regulation of oxidative stress and other mechanisms, thus influencing aging-related genes to delay cellular aging (76, 77). Additionally, SIRT1 plays a key role in regulating cellular energy status and metabolic pathways, and participates in DNA repair processes, enabling tumor cells to survive under drug-induced stress (78–80). Furthermore, mitophagy amplifies tumor cell drug resistance by modulating fatty acid oxidation and ATP production (81, 82).

Studies have shown that cisplatin can cause mitochondrial damage in tumor cells, activating mitophagy, lowering ROS levels, and sustaining ATP production, which increases the cells’ resistance to cisplatin toxicity (83, 84). Likewise, doxorubicin can induce autophagy via mitochondrial damage, helping tumor cells evade drug-induced apoptosis (85).

In mouse models of colorectal cancer, inhibition of mitophagy significantly reduced tumor cell resistance to chemotherapy and increased their sensitivity to chemotherapeutic agents (86). Clinical studies have shown that specific inhibition of SIRT1 can notably enhance the efficacy of chemotherapy and effectively reverse resistance in lung cancer treatment (87). Furthermore, studies in some colorectal cancer patients have revealed that enhanced mitophagy is closely associated with chemotherapy resistance. Therefore, inhibiting mitophagy may represent an effective strategy for overcoming drug resistance (88).

### 5.4 Cell migration and invasion

Similarly, tumor cell migration and invasion are critical processes in tumor metastasis. Mitophagy promotes tumor cell migration and invasion by regulating the activity of related enzymes and signaling pathways. GTPases such as RhoA, RhoB, and RhoC regulate cell morphology, adhesion, and migratory ability, and are involved in tumor metastasis (89, 90). Mitophagy facilitates tumor cell migration and invasion by modulating the activity of Rho GTPases and remodeling the cytoskeleton (91).

Furthermore, when mitophagy is enhanced, the Wnt signaling pathway is activated, promoting tumor cell adhesion and motility, thereby increasing invasiveness (92, 93). In a lung cancer model, inhibition of mitophagy significantly reduced tumor cell migration and invasion (94). In a liver cancer model, promoting mitophagy regulates the activity of Rho GTPases, enhancing the invasive ability of tumor cells (95).

### 5.5 Regulation of the tumor microenvironment

The tumor microenvironment (TME) plays a central regulatory role in tumor initiation and progression. The TME consists of tumor cells, fibroblasts, immune cells, vascular endothelial cells, the extracellular matrix, and various soluble factors. Its internal components influence tumor behavior through continuous and dynamic interactions (96). Studies have shown that the TME not only provides structural support and biochemical cues for tumor cells, but also sustains tumor growth by regulating inflammation, promoting angiogenesis, inducing immunosuppression, and driving invasion and metastasis (97). Further research indicates that ammonia, as an important metabolic regulator, can directly influence the immune microenvironment and thereby modulate anti-tumor immune function (98).

Mitophagy also regulates the metabolic state of immune cells, influencing their functIn GITs, the tumor microenvironment plays a pivotal role.

Mitophagy not only enhances the resistance of tumor cells by modulating the interactions between immune cells and tumor cells, but also prevents the excessive activation of immune cells such as dendritic cells and macrophages, thereby facilitating immune evasion by the tumor (16, 99, 100).

Mitophagy plays an important role in tumor immune escape, involving multiple signaling pathways. For instance, PINK1/Parkin-mediated mitophagy helps maintain mitochondrial homeostasis, limits ROS accumulation, and suppresses immune cell activation and the release of inflammatory factors. The mTOR/AMPK pathway influences anti-tumor immune responses by modulating the metabolic state and effector functions of T cells and macrophages. Additionally, the NF-κB/STAT3 pathway can dampen immune responses and promote tumor immune escape by controlling the expression of immunosuppressive factors (101, 102). Together, these findings indicate that mitophagy shapes immune cell function and contributes to tumor immune tolerance in the tumor microenvironment through the coordinated regulation of metabolic and signaling networks. In animal studies, blocking mitophagy has been shown to strengthen anti-tumor immune responses and improve the effects of immunotherapy (103). Clinical observations also suggest that tumors with higher mitophagy levels tend to exhibit stronger immune escape capabilities (104).

## 6 The role and challenges of mitophagy in different GITs cells

**Figure 4:**
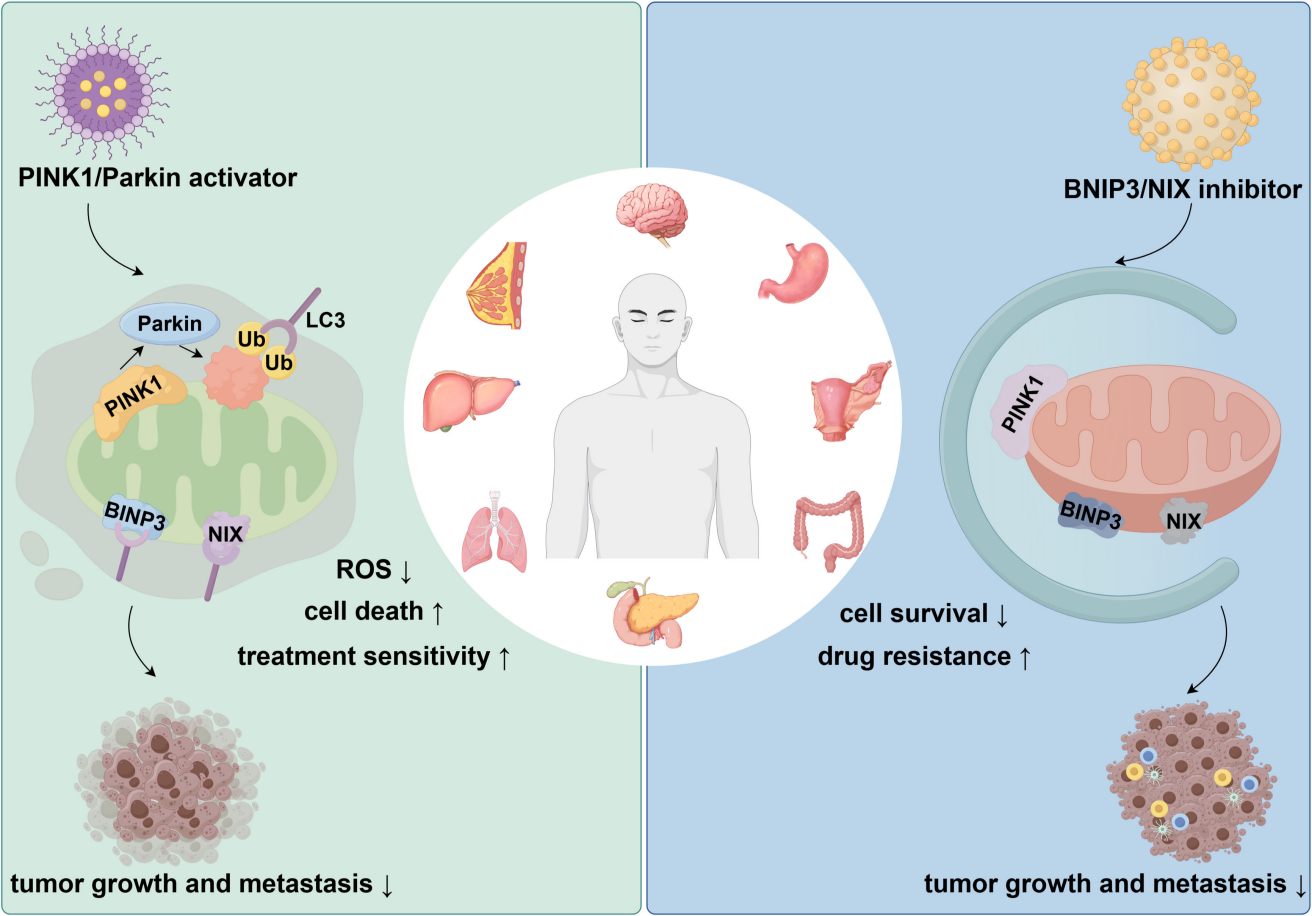
Impact of PINK1/Parkin activator and BNIP3/NIX inhibitor on tumor growth and metastasis. Legend of **Figure 4**: This figure illustrates the contrasting effects of PINK1/Parkin activators and BNIP3/NIX inhibitors on tumor progression. Activation of the PINK1/Parkin pathway increases cell death, reduces ROS levels, and enhances treatment sensitivity, leading to decreased tumor growth and metastasis. Conversely, inhibition of BNIP3 and NIX enhances cell survival and drug resistance, promoting tumor growth and metastasis.

Different tumor cell types, such as gastric epithelial cells, intestinal epithelial cells, and tumor-associated fibroblasts, play distinct roles within the tumor microenvironment. However, they all utilize the same mitophagy mechanisms to collectively regulate key processes of tumor growth, drug resistance, metastasis, and immune escape. For a detailed description of the molecular pathways, mechanisms of action, and animal and clinical trial data, please refer to **Table 2**. As shown in **Figure 4**, the PINK1/Parkin activator enhances treatment sensitivity and reduces tumor growth and metastasis, while the BNIP3/NIX inhibitor promotes cell survival and drug resistance, facilitating tumor progression.

**Table 2:** Mitophagy in different gastrointestinal tumor cell types: mechanisms, roles, and challenges.

Cell type	Key molecules/pathways	Mechanism of action	Models	References
Gastric Epithelial Cells	PINK1/Parkin	Maintains metabolic balance, eliminates damaged mitochondria, prevents ROS accumulation, promotes proliferation and survival	Gastric cancer patient models; Mouse models	(105–108)
Intestinal Epithelial Cells	PINK1/Parkin	Maintains ATP supply, regulates ROS levels, promotes tumor cell proliferation and survival	Colorectal cancer patient models; Mouse models	(109–112)
Tumor-Associated Fibroblasts	BNIP3, NIX	Regulates metabolism and energy supply, promotes tumor growth and metastasis	Mouse models; Rat models	(113, 114)
Macrophages	PINK1/Parkin, SIRT1	Regulates immune response, suppresses oxidative stress, supports immune function and promotes immune escape	Mouse models; Human tumor samples	(53, 115–117)
T Cells	PINK1/Parkin, AMPK, Sirtuins	Maintains immune function, enhances immune surveillance, suppresses immune escape	Colorectal cancer patient models; Mouse models	(118–121)
Dendritic Cells	PINK1/Parkin, Sirtuins	Enhances antigen presentation, initiates immune response, suppresses immune tolerance	Mouse models	(122–124)
Tumor Endothelial Cells	BNIP3, NIX, Rho GTPases	Maintains vascular stability and growth, supports tumor angiogenesis	Renal cancer mouse models	(125–127)
Tumor Stem Cells	PINK1/Parkin, SIRT1	Maintains survival, proliferation, drug resistance, adapts to tumor microenvironment stress	Clinical patient models; Mouse models	(128–131)

### 6.1 Therapeutic potential of mitophagy

Mitophagy exhibits a classic “double-edged sword” effect in the pathogenesis and treatment of GITs. On one hand, mitophagy provides a survival advantage to tumor cells by removing damaged mitochondria, maintaining cellular energy metabolism, and reducing oxidative stress levels. Under stress conditions such as chemotherapy and radiotherapy, tumor cells can activate mitophagy through pathways such as PINK1/Parkin, BNIP3, or FUNDC1 to counteract the damage caused by drugs and radiation, leading to the development of resistance. Moreover, excessive reliance on mitophagy may impair immune cell function and promote tumor immune escape.

On the other hand, mitophagy can exert anti-cancer effects in certain contexts. In the early stages of tumorigenesis, moderate mitophagy helps eliminate potentially oncogenic organelles, reduces DNA damage, and mitigates the risk of genetic mutations, thus halting the carcinogenesis process. Furthermore, certain drugs or natural products can induce energy crisis and cell death by excessively activating mitophagy, thereby directly inhibiting GITs cells. For example, melatonin induces tumor cell apoptosis by activating mitophagy and promoting ROS accumulation (132). Bafilomycin, when combined with chemotherapeutic agents, enhances tumor cell death by inhibiting the fusion of autophagosomes and lysosomes, producing a synergistic effect (133). Nuciferine inhibits mitophagy by blocking autophagosome–lysosome fusion, thereby increasing the sensitivity of tumor cells to chemotherapy-induced death (134). Ketoconazole and mitochondria-targeted metformin (Mito-Metformin) trigger mitophagy through regulation of the PINK1/Parkin pathway, leading to suppressed tumor cell proliferation (135, 136). In addition, andrographolide, a natural antioxidant, and EGCG, a polyphenolic catechin from green tea, show strong antitumor activity in colon cancer (137).

More importantly, regulating mitophagy in immune cells can restore their metabolic activity and effector functions, enhancing the anti-tumor effects of immunotherapy. Therefore, mitophagy in GITs may serve both as a “protective shield” promoting tumor survival and as a “nemesis” that induces tumor cell apoptosis and boosts immune effects. The key challenge in the future is how to precisely modulate mitophagy in different clinical settings to optimize GITs treatment strategies.

Mitophagy-based treatment strategies have demonstrated considerable effectiveness in GITs. Combining these approaches with conventional therapies could further optimize treatment outcomes. Research indicates that when tumor cells activate mitophagy, their resistance to standard treatments is significantly heightened (138). Therefore, inhibiting mitophagy may serve as an effective strategy to overcome tumor drug resistance. Combining mitophagy inhibitors with chemotherapy or radiotherapy can strengthen the cytotoxic effects of these treatments, help reverse resistance, and ultimately improve therapeutic efficacy and patient survival.

Immunotherapy has brought notable progress to the treatment of gastrointestinal tumors, but its effectiveness is still limited by the complex regulation of the immune microenvironment. Mitophagy, a key process that sustains the metabolic balance and functional activity of immune cells, is now gaining attention as a potential target for immunotherapy. Appropriate levels of mitophagy help remove damaged mitochondria and maintain T-cell energy metabolism and cytotoxic activity; however, disruption of autophagy balance can result in metabolic reprogramming disorders and immune exhaustion, which weaken the anti-tumor immune response. For example, several studies have shown that activation of the PINK1/Parkin pathway supports mitochondrial quality control in CD8^+^ T cells and enhances their ability to kill tumor cells (139). In contrast, excessive mitophagy can lead to energy depletion in antigen-presenting cells and increased PD-1 expression, thereby promoting immune escape (140, 141). Moreover, tumor cells may evade immune recognition by upregulating mitophagy receptors such as BNIP3 and FUNDC1, leading to a state of immune tolerance (142, 143). Previous studies have also indicated that regulating mitophagy in combination with immune checkpoint therapy can significantly improve therapeutic outcomes in tumor treatment (144). Therefore, targeting mitophagy may offer new perspectives for improving the response to immunotherapy.

### 6.2 Challenges of mitophagy in clinical applications

Currently, clinical research on mitophagy in gastrointestinal tumors is still in its early stages, with most studies relying on *in vitro* experiments and animal models. Although a small number of observational studies have explored its role in tumor prognosis and immune therapy response, there is a lack of systematic interventional studies and large-scale clinical trials.

Although mitophagy is increasingly recognized for its potential in treating GITs, significant obstacles remain in its clinical application. As a self-regulatory mechanism within cells, the activity of mitophagy must be carefully balanced. Both excessive suppression and overstimulation can lead to undesirable effects, particularly regarding cellular energy supply and metabolic stability. While some mitophagy modulators have shown promising results in early studies, a comprehensive assessment of their long-term effects, management of potential side effects, and ensuring their clinical safety are essential for successful implementation in clinical practice.

## 7 Conclusion and future perspectives

Mitophagy plays a crucial role in the development, progression, drug resistance, and immune evasion of GITs. By regulating mitophagy, tumor cells can adapt to adverse conditions such as hypoxia and nutrient deprivation, maintain metabolic stability, and promote proliferation and metastasis. Additionally, mitophagy influences tumor immune evasion pathways by modulating immune cell activity, thereby facilitating immune escape. Although mitophagy offers a promising theoretical framework for cancer treatment, significant challenges remain in its clinical translation, particularly regarding drug safety, individual treatment variations, and efficacy validation. Future research should focus on the precise regulation of mitophagy, investigate its potential synergistic effects with other therapies, and advance its clinical application in treating GITs.
